# Effects of Combining Medication and Pivotal Response Treatment on Aberrant Behavior in Children with Autism Spectrum Disorder

**DOI:** 10.3390/children5020019

**Published:** 2018-01-30

**Authors:** Mohammad Rezaei, AliReza Moradi, Mehdi Tehrani-Doost, HamidReza Hassanabadi, Reza Khosroabadi

**Affiliations:** 1 Institute for Cognitive Science Studies (ICSS), 16516-56717 Tehran, Iran; mohammad.rezaei@umsha.ac.ir (M.R.); tehranid@sina.tums.ac.ir (M.T.-D.); 2 Pediatric Developmental Disorder Research Center, Hamadan University of Medical Sciences, 65178-38695 Hamadan, Iran; 3 Department of Clinical Psychology, Kharazmi University, 37551-31979 Tehran, Iran; 4 Department of Educational Psychology, Kharazmi University, 37551-31979 Tehran, Iran; hasanabadi@khu.ac.ir; 5 Institute for Cognitive and Brain Sciences, Shahid Beheshti University GC, 19839-69411 Tehran, Iran; r_khosroabadi@sbu.ac.ir

**Keywords:** medication, behavioral therapy, autism spectrum disorder

## Abstract

The purpose of this study was to investigate the effects of combined risperidone (RIS) and pivotal response treatment (PRT) on children with autism spectrum disorder (ASD). A total of 34 children diagnosed with ASD according to the Diagnostic and Statistical Manual of Mental Disorders, fifth edition (DSM-V) (mean age of 12.36 years) were randomly assigned to either of two groups; the first group (*n* = 17) received combined PRT–RIS while the second group (*n* = 17) received RIS only. Behavioral problems were evaluated with the Aberrant Behavior Checklist (ABC), whereas global improvement (GI) was measured with the Clinical Global Impressions (CGI). Assessment of ABC was performed before intervention, after intervention (12 weeks), and following 3 months of the intervention (follow-up). Total ABC scores were seen to decrease in both groups after 3 months, as compared with the scores prior to the interventions. Also, in both groups, mean scores of behavioral problems after the intervention were not significantly different from those prior to the intervention, in all subscales but the inappropriate speech (*p* < 0.001). However, both groups showed significant differences in mean scores of ABC subscales in both of the post-intervention evaluation stages. It was concluded that the combination of behavioral and drug interventions can further improve behavioral problems, ultimately improving patient’s communication and social skills.

## 1. Introduction

Autism spectrum disorder (ASD) is a neurodevelopmental disorder characterized by persistent deficits in social communication and social interaction across several situations and by restricted, repetitive patterns of behavior, interests or activities [1]. People with autism often have other problems, such as hyperactivity, impulsiveness, tantrum, aggression, and self-injury, which affect their daily activities, including communication, social development, and acquisition of educational skills [2,3].

The exact number of people with autism in Iran is not reported, but some studies have shown that the prevalence of this disease in Iran is increasing and its frequency is estimated to be 6 in 10,000 [4].

In principle, there is no specific treatment for autism, and various interventions been proposed to improve associated communication and behavioral problems, such as drug interventions, behavioral interventions, cognitive rehabilitation interventions, and non-invasive treatments [5]. The use of drug therapy, including psychostimulants, antipsychotics, and serotonin reuptake inhibitors, is very common in people with autism. It seems that these drugs generally treat the common symptoms and problems associated with ASD [6]. Several research findings have placed an emphasis on effectiveness of atypical antipsychotics on treating behavioral problems such as hyperactivity, self-injury, aggression, resistance to change, and stereotypes [6]. However, there are no specific pharmacological interventions for treatment of core symptoms of ASD [7]. One of the most commonly prescribed drugs for ASD is risperidone, which has the approval of the Food and Drug Administration (FDA) for children with autism. Risperidone is an antipsychotic and post-synaptic blocker of serotonin and dopamine receptors, and its short-term effect on severe behavioral problems in children with autism has been confirmed in several studies; the most noticeable effects of this drug seem to be in the areas of hyperactivity, aggression and repetitive behaviors [8,9], though, there has been little evidence on effectiveness of this drug on improvement in communication and social functions, language or cognition skills [10].

Pivotal Response Treatment (PRT) is a behavioral intervention that has been effective in improving communication skills, increasing social interactions, and reducing the behavioral problems of children with autism [11,12,13,14]. This approach targets pivotal behaviors such as motivation, initiation, response to multiple symptoms, management and empathy [15]. Studies have indicated that PRT is more effective than the adult-directed applied behavior analysis (ABA) intervention [12]. The use of favored stimulus items and activities, rather than artificial stimuli and the presentation of the teaching within the context of natural interactions during the PRT sessions may have created more interest in the teaching sessions, thereby resulting in lower levels of disruptive behavior [13].

Despite the adequacy of risperidone in treating some behavioral problems, it has no significant effect on learning, adaptive behaviors, and other major problems in children with autism, and the symptoms will be relapsed upon stopping its intake [16]. So, the question is whether or not implementing an appropriate behavior therapy program can help improve these problems and reduce behavioral problems?

The main objective of this study was to investigate the effect of medication and behavioral interventions on reducing the behavioral problems in children with autism, and to compare the results with the effects of medication alone. Findings of this study will help professionals involved in the process of treating children with ASD in selecting the best model for intervention.

## 2. Materials and Methods

According to previous studies, with a standard deviation of 2, an exponent of 80%, and a confidence level of 95%, minimum sample size required in each intervention group was calculated to be 18 individuals. Therefore, a total of 36 patients were selected via convenient sampling and assigned randomly and equally to either of two groups, one receiving combined pivotal response treatment and risperidone (PRT–RIS) treatment and the other receiving RIS alone for 12 weeks. Both interventions (PRT + RIS and RIS) were started simultaneously and the participants did not receive any psychosocial therapies during the trial.

Used as research instrument were personal information questionnaire, aberrant behavior checklist (ABC) [17], serial subscale of Persian Aphasia Battery Test [18], and PRT manual. Personal information questionnaire included demographic information, academic background, hearing and vision statuses, and existence or absence of bilingualism.

**Aberrant Behavior Checklist (ABC):** The checklist includes 58 questions designed to assess the presence and severity of maladaptive behaviors in people with developmental disabilities. This tool evaluates five categories of behavioral disorders, three of which are the main defects in autism: lethargy, stereotypical behavior, and inappropriate speech, with the other two being related to hyperactivity/restlessness and irritability. Validity and reliability of this instrument have been confirmed in several studies [17].

**Clinical Global Impressions-Improvement Scale (CGI-I):** The scale is a three-point observation which evaluates the severity of the disease (CGIS), overall improvement (CGIC), and therapeutic response. CGIS and CGIC are more widely used in clinical studies and centers. The questionnaire has been developed based on a 7-point Likert scale [19].

Initially, the families of children with autism were asked to study and sign the research information and consent form. During the interview with each subject’s father or mother and with the help of the items contained in the child’s record, the subject’s personal information form was completed. Then, the parents were provided with the questions extracted from PRT guideline, asking about the child’s favorite materials and activities, so as to capture specific characteristics of each child. Then, tailored to the motivational interests and methods of each child, his/her special treatment plan was designed. Afterwards, the 36 subjects were selected and matched in terms of age, sex and length of utterance, in a pairwise fashion. Then, they were randomly assigned to either of two intervention groups. One of the groups received combined PRT–RIS treatment and the other received RIS only. Before the intervention, children’s behavioral status was evaluated by the ABC questionnaire, and changes in overall functioning were assessed by Clinical Global Impressions-Improvement score (CGI-I) rated by a clinician who remained blind to treatment assignment. Behavioral interventions were conducted over a 12-week period. Families of two children could not continue to participate in this study due to financial and social problems.

### Interventions

**Risperidone Dose Schedule:** At the beginning of the study, the children weighing less than 45 kg received a dose of 0.5 mg of risperidone at bedtime, and the dosage continued until twice a day 0.5 mg for 7 days. The dosage was gradually increased at a rate of 0.5 mg per step to reach a maximum of 2.5 mg per day on day 29. Children weighing more than 45 kg started at higher doses, reaching a maximum dosage of 3.5 mg per day by day 29 [20].

After week 12, the medication was reduced to 75% of the maintenance dose for week 13, then to 50% for weeks 14 through 15, to 25% for week 16, and to 0 for weeks 17 through 20. After week 20, the parents were encouraged to continue the treatment arbitrarily. Subjects were monitored on a weekly basis for signs of relapse (i.e., a score of Much Worse or Very Much Worse on the CGI-I scale).

**Pivotal Response Treatment [1]:** The PRT was accomplished according to its published manual titled as PRT: Using Motivation as a Pivotal Response [21]. In this intervention, items were selected based on the child’s favorites rather than the clinician accidentally choosing a stimulus item. The task was mixed so that the reward was presented both for responses that were previously learned (herein referred to as shorter utterance) propagated with rewards for new acquiesced tasks (herein referred to as longer utterances). The reinforcement was eventuality expanded in such a way that the child was reinforced if he/she either imitated the precise accurate response or a succeeding estimation or made any clear verbal try to respond. Rather than the child being reinforced with eatables and admiration, the child was reinforced with the time for play with the educational stimulus, paired with verbal admiration.

Forty-five-minute treatment sessions were carried out three times a week over a 12-week-period (a total of 27 h for each participant). All intervention sessions were conducted in a 10′ by 15′ room using a one-to-one teacher–child format. Used for reducing aberrant behavior, PRT method was implemented according to our previous study [13]. The treatment rooms contained a table, chairs and stimulus materials. The PRT providers were speech/language pathologists (SLPs) who had a minimum of 5 years of experience in teaching children with autism. Prior to start of the study, the performers contacted individuals with experience in the interventions and were provided with particular practices for their specific condition. Parents were informed that their children would receive speech and language therapy, but they remained blinded to the detailed goal behavior (increasing Mean Length of Utterance (MLU)). The parents were not present during the intervention sessions. For all children, similar goal behaviors were set: increasing the child’s MLU using recast procedures [22]. Results of our previous study have indicated further effectiveness of PRT in improving targeted language acquisition (MLU) and untargeted areas (disruptive behavior). PRT uses as tools the child-preferred items and activities.

Throughout the study, each child was observed at least four times and scored for Fidelity of Implementation (FoI) by the first author. FoI was scored for a total of 10 min in 1 min intervals, and each of the seven points were scored as correct (+) or incorrect (−) according to each of the variables outlined in Table 1. As noted in Table 1, the SLPs had to attract the child’s attention, offer an obvious choice, and arrange conditional outputs. FoI for treatment providers ranged from 80% to 90% and never fell below the required threshold of 80%.

After the 12 weeks, the ABC test was completed. Also, this test was taken 3 months after the interventions to check the rate of symptom recurrence. Following standardized instructions, an independent rater (A fourth-year Ph.D. student in behavioral sciences) asked parents to complete the ABC rating scale. Finally, the scores of the subjects were analyzed before, immediately after and 3 months after the intervention.

**Side effects:** Side effects were recorded throughout the study and were assessed using a checklist (a 34-item checklist including a wide range of adverse events) by an independent rater during weeks 4, 8 and 12. This was done through questioning. The behavior ratings and the side effects were performed by independent raters.

**Statistical analysis:** All data was analyzed with SPSS 16.0 software (copyright 1993–2007 Polar Engineering & Consulting, Kenai, AK, USA). Descriptive data were analyzed with the so-called *t*-test. Descriptive statistics used herein included mean, standard deviation, percentages and frequencies. Independent *t*-test was used to compare scores at the baseline between groups and repeated measures ANOVA was used to compare the data concerning scores before, immediately after, and 3 months after treatments for both groups. Also, analysis of covariance (ANCOVA) test was used to compare the groups in scores immediately after treatments.

## 3. Results

Of the total of 34 participants who completed this study, 65% were males and 35% females. Analyses of demographic data by independent *t*-test showed that the mean age and length of utterance had no significant difference between RIS–PRT and RIS groups, and the age distribution (*p* = 0.911) and mean length of utterance (*p* = 0.212) were similar in both groups prior to the start of intervention (Table 2).

Then, the ANCOVA test was used to check for the effect of the test before intervention, and mean score of behavioral problems in the two groups after the intervention was evaluated. As shown in Table 3, there was no significant difference in all subscales other than inappropriate speech and social withdrawal (*p* < 0.001). Indeed, the RIS–PRT group showed significantly more improvement in inappropriate speech and lethargy 3 months after the intervention.

An independent sample test showed no significant difference between the two groups in CGI score (*p* = 0.786) after intervention.

Also, the repeated measures ANOVA test was used to compare mean scores of ABC test in the three phases, namely before, immediately after, and following 3 months of the intervention. As shown in Table 4, both groups showed a significant difference between the mean scores of ABC subscales in the three stages of evaluation. Also, the results of inter-group analysis showed a significant difference between the two groups only in social withdrawal (lethargy) behavior sub-scale (*p* = 0.001) in the follow-up phase (3 months after the interventions). There was no significant difference between the two groups in other sub-scales of the behavioral problems (irritability, stereotypic, hyperactivity and inappropriate speech) (*p* = 0.395, 0.171, 0.241, and 0.926, respectively).

Frequencies of adverse events are summarized in Table 5, showing no significant difference between the two treatment groups in this respect.

## 4. Discussion

To the best of our knowledge, no study has been reported on the effect of combined PRT–RIS. In this research, after three months of intervention, the behavioral problems of the subjects decreased significantly in both intervention groups. The results of this study showed that drug therapy alone may not impose any significant contribution to reducing inadequate speech and social problems of autistic children, but once combined with a response-based treatment, the drug therapy can reduce speech problems and improve social skills. Therefore, it can be expected that behavioral interventions may have a greater effect on communication and speech ability. By choosing the child’s favorite items and activities and the use of natural promoters in the response-based treatment, the child’s motivation toward and his/her desire to initiate an interaction increase [11,23]. Results of the present study were consistent with those of the study by Aman et al., who suggested that the combination of risperidone and behavioral intervention (rather than drug intervention alone) is more effective for reducing behavioral problems [24].

Various studies have shown that risperidone has a significant effect on the reduction of stereotypic behaviors, hyperactivity and irritability [25]. Similarly, the results of this study indicated attenuation of the mentioned behaviors due to the effect of risperidone in both intervention groups. However, effectiveness of this drug on these behaviors is not a new and surprising result, because in previous studies, haloperidol has also had similar effects on the reduction of stereotypic behaviors [26].

Also, in line with the results of this study, other studies showed that risperidone alone does not have a significant effect on speech and lingual skills. As with the results of this study, risperidone can prepare a child with autism to participate in social interactions, thereby improving social withdrawal by reducing other behavioral problems such as bad temper, tantrum, stereotypic behavior and hyperactivity [20].

Also, in this study it was found that, upon stopping the treatment, the child had his/her behavioral problems returned, but the rate of recurrence in the group receiving the combined treatment was lower. On the other hand, after discontinuing the treatment, there was no significant difference between the two groups of drug only and combined interventions in other behavioral problems except for lethargy (social withdrawal) behaviors, which indicated the necessity of the continuation of the combined treatment for improving the behavioral problems of children with autism. So, according to other studies, despite the adequacy of risperidone in the treatment of some behavioral problems, this drug has no significant effect on learning, adaptive behaviors and other major problems in children with autism, and the recurrence of symptoms will be observed upon stopping the drug treatment [15]. Also, developing an appropriate behavioral therapy program may help improve these problems while reducing behavioral problems and the rate of problem relapse to some extent. Some studies have shown that children with verbal communication deficits reported more behavioral problems than verbally healthy children [27]. Accordingly, it seems that there is an inverse relationship between behavioral problems, such as irritability, and communication deficits, such as inappropriate speech.

In general, it can be concluded that risperidone reduces behavioral problems, thereby preparing the child for engaging in social interactions. On the other hand, behavioral interventions can increase efficacy of the drug and increase the child’s readiness to learn speech and language skills. Therefore, a combination of behavioral and drug interventions can improve behavioral problems in a faster and more appropriate fashion, ultimately improving their communication and social skills.

One of the limitations of the study was the low number of subjects, so that the results of this study cannot be absolutely generalized, in which risperidone alone was not sufficient for reducing speech problems and increasing language skills. Accordingly, it is recommended to examine the effect of combining other behavioral interventions with other drugs in future studies. This study was conducted on a sample of children with ASD but no intellectual disability (IQ > 70). Thus, findings might be different in children with lower IQ (intelligence quotient).

## Figures and Tables

**Table 1:** Descriptions for fidelity of implementation. Adapted from [1].

**Child Attending**
The speech/language pathologists (SLP) must attract child’s attention before provoking a chance.
**Obvious Chance**
The question/education/opportunity to respond must be clear and suitable for the task.
**Child Choice**
The SLP should follow the child’s choice with tasks and actions. If child is not showing interest in the present task, SLP should effort to alter the practice.
**Keep Tasks**
The SLP should combine tasks the child can already complete with new tasks.
**Conditional**
Reinforcement must be depending on child’s behavior. The SLP’s response must be contingent upon the child’s response.
**Natural**
Reinforcement should be natural or right associated to the favorite behavior.
**Depending on Challenges**
Any target-directed effort to respond to questions, guidelines, or chances should be reinforced.

**Table 2:** Baseline Characteristics.

Variable	RIS Group (*n* = 17) Mean *±* SD	PRT + RIS Group (*n* = 17) Mean *±* SD	*p*
Age	148.94 ± 29.41	144.70 ± 37.58	0.911
MLU	3.54 ± 0.60	3.23 ± 0.80	0.212
Age of onset	28.72 ± 4.21	29.68 ± 5.80	0.588
Weight	44.35 ± 12.31	43.82 ± 14.93	0.799
Irritability	2.72 ± 23.23	4.86 ± 23.05	0.897
Lethargy/social withdrawal	5.16 ± 30.41	28.52 ± 5.00	0.288
Hyperactivity/noncompliance	8.65 ± 22.94	8.75 ± 19.58	0.27
Stereotypic behavior	3.05 ± 17.23	4.45 ± 15.05	0.107
Inappropriate speech	1.76 ± 9.00	1.67 ± 9.94	0.285

RIS: Risperidone; PRT: pivotal response treatment; SD: standard deviation; MLU: Mean Length of Utterance.

**Table 3:** MLU and ABC subscale scores for RIS and RIS plus PRT groups after treatment.

Variable	RIS Group (*n* = 17) Mean ± SD	PRT + RIS Group (*n* = 17) Mean ± SD	Mean Difference	*p*
MLU	0.591 ± 3.60	0.832 ± 4.23	0.930	≤0.0001
Irritability	2.10 ± 13.76	4.80 ± 13.41	0.247	0.806
Lethargy/social withdrawal	4.84 ± 26.94	6.49 ± 20.88	4.299	0.001
Hyperactivity/noncompliance	6.39 ± 14.64	6.05 ± 12.88	0.431	0.631
Stereotypic behavior	2.34 ± 9.41	4.52 ± 8.05	0.002	0.999
Inappropriate speech	5.171 ± 94	4.471 ± 84	1.514	0.001

ABC: Aberrant Behavior Checklist.

**Table 4:** Mean ± SD of the Two Treatment Groups on the Subscales of ABC at phase three.

Variable	Baseline Mean ± SD	Endpoint Mean ± SD	Follow-Up Mean ± SD	*p*
**Irritability**				
RIS	2.72 ± 23.23	2.10 ± 13.76	1.39 ± 17.76	≤0.0001
RIS + PRT	23.05 ± 4.86	13.41 ± 4.80	16.82 ± 4.27	
**Lethargy/social withdrawal**				
RIS	30.41 ± 5.16	26.94 ± 4.84	27.94 ± 4.86	≤0.0001
RIS + PRT	28.52 ± 5.00	20.88 ± 6.49	21.35 ± 5.33	
**Hyperactivity/noncompliance**				
RIS	22.94 ± 8.65	14.46 ± 6.39	18.11 ± 7.00	≤0.0001
RIS + PRT	8.75 ± 19.58	6.05 ± 12.88	15.35 ± 6.48	
**Stereotypic behavior**				
RIS	17.23 ± 3.05	9.41 ± 2.38	12.47 ± 2.06	≤0.0001
RIS + PRT	15/05 ± 4.45	8/05 ± 4.52	10/94 ± 4.00	
**Inappropriate speech**				
RIS	9.00 ± 1.76	5.17 ± 1.94	6.58 ± 1.87	≤0.0001
RIS + PRT	9.94 ± 1.67	4/47 ± 1.84	6.64 ± 1.72	

**Table 5:** The frequencies of adverse events in two groups.

Variables	RIS Group (*n* = 17) N (%)	PRT + RIS Group (*n* = 17) N (%)	*p*
Increased appetite	4 (23.5)	3(17.6)	0.671
Nausea	2 (11.8)	2(11.8)	1
Urinary problems	3 (17.6)	2(11.8)	0.682
Increased weight	2 (11.8)	2(11.8)	1
Sedation	1 (5.9)	1(5.9)	1
Dizziness	1 (5.9)	2(11.8)	0.545
Constipation	3 (17.6)	3(17.6)	1

N (%): number (percentage).
